# Applying critical discursive psychology to health psychology research: a practical guide

**DOI:** 10.1080/21642850.2020.1792307

**Published:** 2020-07-23

**Authors:** Abigail Locke, Kirsty Budds

**Affiliations:** a Division of Psychology, School of Social Sciences, Faculty of Management, Law & Social Sciences, University of Bradford, Bradford, UK; b Division of Psychology, School of Social Sciences, Leeds Beckett University, Leeds, UK

**Keywords:** Critical discursive psychology, baby led weaning, qualitative, critical health psychology, health communication

## Abstract

This paper outlines a qualitative methodological approach called Critical Discursive Psychology (CDP), considering its applicability to health psychology research. As applied to health psychology, the growth of discursive methodologies within the discipline tends to be located within a critical health psychology approach where CDP and others enable a consideration of how wider societal discourses shape understandings and experiences of health and illness. Despite the increasing usage of CDP as a methodology, little has been written on the practical application of the method to date, with papers instead focusing on the theoretical underpinnings of a CDP approach. This paper seeks to address that gap and offers a step by step guide to the key principles and analytic stages of CDP before giving a worked example of CDP applied to a health topic, in this case ‘baby-led weaning’ (BLW). As we discuss, a key strength of CDP, particularly in relation to health psychology, is in its attempts to understand both macro and micro levels of data analysis. By doing so it offers a nuanced and richer understanding of how particular health topics are working within context. Therefore, CDP is a readily applicable analytic approach to contested and complicated topic areas within health research.

## Introduction

This paper focuses on the applicability of Critical Discursive Psychology (CDP) to research within health psychology. As yet, despite the growth of CDP papers in recent years (e.g. Budds, Locke, & Burr, 2016; Locke, 2015; Locke & Yarwood, 2017; Wetherell & Edley, 2014) since Edley’s (2001) chapter on outlining a critical approach to discursive analysis, there has been a dearth of practical guides on how to actually conduct a CDP analysis. This paper begins by introducing and locating CDP within its wider discursive backdrop, outlining key principles of the methodology. Building on previous work that began to map the steps of CDP (Budds, 2013: Budds, Locke, & Burr, 2017), it then moves to a guide on how to conduct a CDP analysis before offering a worked example applied to a health topic – baby-led weaning (BLW). Please see Locke (2015) for further examples of CDP applied to BLW and infant feeding.

CDP is one of a variety of approaches that sits under the umbrella of discursive research. Discursive methodologies are well established across the Social Sciences. With regards to the discipline of Psychology, their initial usage and development came in late 1980s through Potter and Wetherell (1987) as one of a growing number of qualitative research methods as part of the ‘discursive turn’ that followed the crisis in social sciences (Harré, 2003). Under the umbrella of the discursive methodologies sit a number of variants including Discourse Analysis (Potter & Wetherell, 1987), Foucauldian Discourse Analysis (Willig, 2013), Discursive Psychology (Edwards & Potter, 1992; Wiggins & Potter, 2017) and Critical Discursive Psychology (Wetherell, 1998; Wetherell & Edley, 2014). However, what these methodologies generally have in common is their social constructionist epistemology which demands that rather than being viewed as an accurate or true representation of people’s thoughts or feelings, language is considered to construct social reality (Burr, 2015). As such, discourse becomes the central focus of investigation.

Critical Discursive Psychology (e.g. Wetherell, 1998, 2015; Wetherell & Edley, 2014) is a form of discursive analysis that embodies principles from both wider (conversation analytically inspired) discursive psychology (e.g. Edwards & Potter, 1992; Wiggins & Potter, 2017) and post-structuralist Foucauldian-inspired Discourse Analysis (e.g. Arribas-Ayllon & Walkerdine, 2008; Willig, 2013). In this paper, we will explain how in Critical Discursive Psychology, these two approaches, often seen as opposing, can come together, before moving on to outline the core principles of these approaches and to consider some examples of how they have been applied to health psychology topics. We will then make a case for what Critical Discursive Psychology (CDP) offers to health psychology research. We begin by providing a brief introduction to Foucauldian Discourse Analysis (FDA) and Discursive Psychology (DP).

### Foucauldian discourse analysis

Often considered to be at either end of the spectrum of discursive methodologies, both Foucauldian Discourse Analysis and Discursive Psychology have successfully been applied to health research and have become popular approaches in psychology more widely. Foucauldian Discourse Analysis (FDA) is an approach to discourse analysis underpinned by post-structuralist traditions and in particular is inspired by the work of French philosopher Michel Foucault. FDA is used to examine the ways in which language and discourse constitute versions of our social reality (Foucault, 1981). A Foucauldian approach tends to involve identifying the discourses that are available to social actors, from which they can make sense of the world around them. For Foucault, discourse is closely bound up with power (Foucault, 1978) and in that sense discourses have implications for practice (what people can do or have done to them) and subjectivity (Willig, 2013). For instance, an example of critical health work from a Foucauldian Discourse Analytic approach includes Gillies and Willig’s (1997) study which examined the wider discursive constructions women used to justify smoking behaviour. Based on these findings they developed some implications for health promotion activities and argued for an approach where the more structural underpinnings of smoking need to be considered and addressed. As we can see with this example, health research inspired by Foucauldian approaches would therefore consider wider discourses of health and illness and the way in which these have implications for practices and subjective experiences.

### Discursive psychology

Meanwhile, other work applies the principles of discursive psychology (DP) to health research (e.g. Wiggins & Potter, 2017). At the heart of DP is a focus on talk and text as part of social practices and a consideration of talk as performative – how people do things with their words. There is a focus on how psychological concepts are invoked and made relevant within interactions (Edwards & Potter, 1992). As an approach, DP has evolved since its inception, shifting from a focus on interviews and interpretative repertoires to a focus on sequential analysis which is informed by conversation analytic principles (Kent & Potter, 2014; Potter, 2012).

Applied to health, Wiggins (2017) considers that DP can address issues such as how individuals orient to particular everyday practices as ‘healthy’ or not, how people seek health advice and how the concepts of health and illness are used within interaction. For example, there is now an established body of research using DP to consider how people manage accountability for either being healthy or coping with illness. Wiggins (2009) used a discursive psychological approach to examine how individuals attending National Health Service obesity services in the UK managed blame for obesity by resisting personal responsibility for weight gain. Focusing on the discourse in group meetings between patients and practitioners, the analysis showed how the patients did this both by denying they had performed any activities which may contribute to blame for weight gain/lack of weight loss and, secondly, by constructing the blame as beyond their direct control. In doing so, Wiggins argues, the individuals conform to individualistic medical understandings of weight management and locate their discourse within the interactional management of blame and weight management. That is, there is an element of ‘moral accountability’ around the ways in which weight is discussed at the local level in these interactions. This micro approach to DP analysis is able to illustrate how these discourses are being constructed at local interactional level. However, when it comes to locating such discourses within social and cultural frameworks, with a DP approach it becomes more difficult as these wider ‘macro’ concerns are outside of the local ‘micro’ interactional level. Please see the debate between Schegloff (1997) and Billig (1999) on context in discourse for a wider discussion of this issue.

### Critical discursive psychology

Whilst these two discursive strands have typically stood apart, Wetherell (1998) began mapping (with Nigel Edley) a discursive approach that focused on elements of post-structuralist discourse analysis (in line with elements of Foucauldian inspired DA) alongside elements of discursive psychology (Edwards & Potter, 1992). (Wetherell, 2007, p. 665). Wetherell’s (1998) paper offered a detailed and comprehensive engagement with both post-structuralist (Foucauldian) and conversation analytic approaches to the study of talk, noting the tensions and possibilities that a combination could offer social (and health) psychology. This potential synthesis was named Critical Discursive Psychology and, building on previous work (Budds, 2013; Budds et al., 2017; Edley & Wetherell, 2001; Wetherell & Edley, 2014), is the methodology that will be outlined in this paper. By combining the micro-analytic elements of Discursive Psychology that pay attention to the ways in which discourse and interaction is a form of ‘social action’ with the wider ‘macro’ elements of socio-cultural and historical contexts of the discourse typically seen in Post-Structuralist approaches, CDP provides a dual-reading of data and can offer a more complete analytic picture of the topic under investigation.

As Edley and Wetherell (2001) note, within CDP:

on the one hand, we try and study how talk is organised as social action in its immediate context, the subject positions in play and the rhetorical and interactional consequences of this organisation, focusing on participants’ orientations to clarify and identify these elements. On the other hand, we assume that talk assumes regular patterns that reveal the shared sense-making resources of a sample or which may be specific to a site, institution or characteristic of a broader social context and historical period. (Edley & Wetherell, 2001, p. 441)

Therefore, for health research, CDP enables us to consider, at a macro level the wider discourses of health and illness, in terms of identifying what these are and what possibilities they open up (and close down) for making sense of health and illness and the subject positions or ‘ways of being’ made available. Additionally, at a micro level, we are able to consider how individuals deploy these discourses locally and in so doing accomplish various social practices. A CDP approach therefore aligns itself with the notion that people are both the products and producers of discourse (Billig, 1991). On one hand, discourse is constructive – discourses shape the possibilities for understanding various concepts and objects in the social world – and therefore shape the possibilities for social practice and subjectivity. For example, we see how discourses of masculinity might shape men’s engagement with health care services (e.g. Seymour-Smith, Wetherell, & Phoenix, 2002). On the other, we are able to consider the way in which individuals have some agency and are able to selectively deploy discourses within interaction in order to accomplish different social practices. In the example above (Wiggins, 2009) we saw how individuals drew on individualised constructions of weight management in order to try to deflect blame and accountability for their weight.

## Three tenets of critical discursive psychology

There are three core principles or tenets of a Critical Discursive Psychological approach that underpin the methodology. Firstly, as noted above, that discourse is both constitutive in that it can shape the various ways in which we are able to make sense of the world around us. Yet it is also constructive – we can actively construct versions of the social world and make use of wider societal discourses within interaction in order to achieve various social practices. We can apply this tenet to the concept of subject positions (Davies & Harré, 1990), which are key to a critical discursive psychological analysis (Budds et al., 2017; Edley, 2001). Subject positions are considered to be particular ‘ways of being’ that are made available within discourse, as Parker (1992, p. 9) notes: ‘a discourse makes available a space for particular types of self to step in’. Therefore, we can consider the ways in which different discourses position individuals, with respect to what subject positions are available. Yet, also, we might consider the way in which individuals are able to position themselves within discourse by either actively taking up or indeed resisting the subject positions that are made available.

The second tenet is that discourse is situated and this situated nature is considered in a number of ways. For CDP theorists, all discourse is indexical, i.e. it is not separable from its context. Therefore we must attend to how data is produced e.g. research interviews, chat room interactions, work place interactions or, as in the case of the data for this piece, newspaper articles. We should also consider, however, how situated discourse is rhetorical. That is, we are producing versions and arguments in a rhetorical way and therefore alternative versions or constructions are always possible. Therefore, as Billig (1991, 1996) suggests, there can be a consideration of what is neglected to be mentioned, as well as what is. A focus of CDP analysis can then be to examine how one version is constructed as plausible whilst others may be discounted (or ignored).

The final key tenet is concerned with the action orientation of talk and management of accountability. That is, CDP asserts that versions of events can be actively constructed through discourse, achieving a variety of social actions. Of particular interest for CDP is the management of accountability – that is, how speakers manage their agency within interactions and can perform a number of different acts such as excusing, justifying or blaming, when retelling their version of events (Edwards, 1997). For example, work from this tradition has seen how women manage accountability for ‘delayed’ motherhood (Budds et al., 2016, p. 2017) or how fathers account for their level of involvement in parenting (Locke & Yarwood, 2017).

## Critical discursive psychology: analytic steps

Discursive psychological methods of data analysis have not typically been produced with a set of stages of analysis that can be followed like a ‘recipe book’ (Gill, 1996), unlike more recent inventions in methods popular in the health sciences. However, we propose there are a number of steps that the developing analysis can be broken down into which move from considering broader constructions within the text to a more thorough consideration of the ‘how’, ‘when’, and ‘why’ of the discourse. In this paper we will outline these steps in the order in which we perform them, illustrating with a worked example.

Prior to analysis, as with all research studies, we would assume that the topic to be studied has been designed and conducted within a CDP framework. That is, in line with Crotty’s (1998) approach to research design, the research question should be congruent with the epistemological, ontological and theoretical framework for the research. For a CDP analysis, typically the epistemology is social constructionism, the ontology is either relativist or critical realist depending on the analyst’s wishes. A relativist perspective would consider language as the medium through which social ‘realities’ are constructed. Whereas, from a critical realist position, language is viewed as constructing social realities, but it is also recognised that these constructions are constrained by material conditions. The theoretical framework is interpretivist, which means there is a focus on understanding and interpreting meaning within the data. Given that this forms part of an advanced methods guide, we would assume some prior knowledge on research design for qualitative research (see Rohleder & Lyons, 2015, for a useful guide). The research questions for a CDP project are often quite open. In the case of the piece here, it would be something like ‘how do newspapers construct baby-led weaning?’

Critical Discursive Psychology can be applied to a variety of different data sources. These include secondary and documentary data, such as official documents or media and newspaper reports; observations of naturally occurring data, such as recordings of ‘real-world’ situations (e.g. self-help groups, medical interactions, helpline recordings, internet chatrooms and forums, and so on); and data which is collected as part of the research process, such as data from interviews or focus groups. When using interview data, critical discursive psychologists have a preference for semi-structured or unstructured interview formats. This is due to focus on the co-production of conversation and knowledge that a CDP approach proposes. However, when used within a CDP framework, it becomes important to analyse the interviews or focus groups in their entirety, as an interaction (e.g. question-answer sequences), instead of focusing entirely on participant responses by way of acknowledging the context accounts are situated within.

Once the data has been collected if it through audio or video recordings then a transcript of the data will need to be produced before the analysis begins. There are different transcription notation methods that exist but the data will need to be at least transcribed verbatim (i.e. word for word). Some CDP analysts use an adaption of the Jeffersonian method (Jefferson, 2004) developed for Conversation Analysis which notes pauses and other intonation in interaction.

The data presented for analysis in this paper came from an analysis of newspapers as the research focused on constructions of ‘baby-led weaning’ in the media (see Locke, 2015, for related work on this topic). To collect the data the media search database *Proquest International Newsstand* was searched using the search terms ‘baby-led weaning’ for all newspapers titles on the database. This produced an initial sample of 585 articles across a number of countries. Once duplicate and other non-related articles were removed, including non-English language articles, a final sample of 78 articles was subject to a Critical Discursive Psychological (CDP) analysis.

### Stage one: familiarisation with the data and initial coding

Once you have your data, it requires a thorough reading and familiarising yourself with the data corpus. Initially the researcher needs to immerse themselves within the data and perform a line by line coding focusing in on what is being said, what categories are being invoked, and when and how they are invoked. We suggest the coding produced at this stage can be both descriptive and interpretative in order to highlight the different ways in which the topic is discussed. The analysis moves from description and ‘noticings’ in the data onto more detailed interpretation as it progresses. For this particular analysis, as it was performed on newspaper articles that had a focus on weaning practices, there were key references to different forms of weaning throughout the whole data corpus. Categories to be identified here could therefore be names of particular practices such as ‘feeding’ and ‘weaning’, or people, e.g. ‘mother’ and ‘baby’. Consider, for example, the extract below.

When the stay-at-home mum did decide to introduce food again she tried baby-led weaning, where children are offered a range of finger foods and they feed themselves what they want to eat. She says: ‘It was down to him what he wanted and the milk was there to fill him. Now he will eat anything. But she says her older two sons, Karlum, aged six, and Jack, aged four, were both weaned at four months and are fussy eaters. (The Sentinel (UK), 25 January 2011)

As we can see in the extract above, there are a number of categories contained in the discourse. These include categories of people and practices, such as ‘stay-at-home mum’ and ‘fussy eaters’, ‘baby-led weaning’, ‘weaning’ and ‘finger foods’. All of these terms are of interest at this initial stage of coding as they all relate to infant feeding practices as the topic of the research project. We will see as we move into stage two, how these initial noticings and categories start to become worked up into a more detailed analysis.

### Stage two: discursive constructions

In this second stage the analysis involves the identification of the constructions of the topic of investigation, building on the coding that has been performed in stage one. The analysis proceeds as the analyst identifies the prevalent themes or ways of talking in the discourse, and how these key words or repeated themes can be grouped together. This stage differs from stages of more thematic approaches, such as Thematic Analysis and Interpretative Phenomenological Analysis (IPA), as the focus here moves away from more factual or experiential concerns about the topic, to an attempt to understand what the words and themes are ‘doing.’ Applied to our example, at this stage, we would consider the different ways in which the discursive object of ‘baby led weaning’ is constructed within the text. We will consider an extract of data from 2008, which appeared in a Canadian newspaper, to illustrate this further.

It’s time to pack up the pea puree and toss the baby rice. No more blending beans, mashing bananas or whipping sweet potatoes. Fed up with rigid timetables for the introduction of first foods, a growing number of parents are giving up on spoon feeding and letting the kids set the pace. (The Globe and Mail (Toronto, Canada), 25 November 2008)

In this extract the caregivers are told to ‘pack up’ the purees and ‘toss the baby rice’, two identifiable traditional weaning methods and foods for infants. It continues with other tried and tested ways of preparing traditional weaning foods of ‘blended’, ‘mashed’ and ‘whipped’ foods. Finally, the baby-led weaning movement is constructed, in contrast, as a way of resisting the ‘rigid’ nature of previous weaning advice and timings, noting the ‘growing number’ of parents who are giving their children agency over their eating practices by letting them ‘set the pace’. Therefore, if we consider this in the light of stages one and two of analysis, we can see how this extract highlights the way in which BLW is constructed as an antidote to, or progression from, traditional infant feeding methods.

### Stage three: interpretative repertoires

In stage three the analysis considers the pervasive constructions of the discursive objects through the identification of interpretative repertoires (Wetherell & Edley, 2014). The analyst begins working through the data in more detail, considering what the invocation of the discursive construction is accomplishing in the context of the interaction. An interpretative repertoire is a recognisable way of describing, framing or speaking about an issue that is identifiable as such. The notion of interpretative repertories was brought into psychology through Potter and Wetherell (1987) as a way of conducting discourse analysis. Questions can be asked such as what kind of reality is being constructed, and, what kinds of constructions are being resisted? Identification of a repertoire is similar in many ways to the identification of a discourse, in that recognisable ways of describing/presenting an issue are identified. However, where the difference lies appears to be around Foucauldian notions of power that sit within many post-structuralist discursive approaches. In that, a discourse commonly has a more pervasive aspect that typically positions different groups in particular ways along power lines. Interpretative repertoires allows for a wider focus on human agency in discursive construction (Edley, 2001). As the analysis develops, we are able to see how different interpretative repertoires are constructed through the talk. In the data presented here, we can see a repertoire of the ‘agentive child’ whereby it is the child who chooses what and how much they will eat from a variety of foods provided by the parent/caregiver and ‘sets the pace’.

The issue of the agentive child with ‘choice’ over their eating habits is a prevalent repertoire throughout the data corpus. Researchers (e.g. Townsend & Pitchford, 2012) have speculated that agentive children are more likely to self-regulate their food intake, and thereby avoid obesity and weight problems in the future. The extract below is from one of the first newspaper articles that discussed this research study (Townsend & Pitchford’s article in the BMJ Open).

Babies may know best when it comes to their future health, according to researchers who found that infants who have more choice over what they eat may be less overweight than their spoon-fed counterparts. Allowing infants to feed themselves from a selection of finger foods from the start of weaning rather than being fed purees may help them regulate their intake. (The Guardian (UK) 7 February 2012)

In the excerpt we can see that the agentive child is clearly constructed. We are told that babies ‘know best’ with respect to their future health and this is determined by them having more ‘choice’ over their eating, in contrast with traditional feeding methods, leading to children learning to self-regulate their food intake. The analysis moves beyond these constructions to see how BLW constructed the child as agentive in their feeding behaviours. The newspaper report notes how the child is given a selection of foods and then will ‘choose’ which of these to explore and eat. This ability for a child to self-regulate is portrayed as something that is inherent in the baby, this is ‘natural’ from birth, through self-regulating their milk feeds, but becomes lost through the practice of spoon-feeding in the weaning process. Therefore, by following BLW, the mother is engaging in a more ‘natural’ type of parenting.

### Stage four: subject positions

Subject positions are a key aspect of post-structuralist analyses and can be considered as ‘ways of being’. Subject positions came from ‘positioning theory’ (Davies & Harré, 1990) which looks at how the writer or speaker is both positioned and positions themselves and others in discourse. In stage four of a CDP analysis, we suggest the focus should turn to the positions that are made available to people through the interpretative repertoires that are in operation. As we saw in stage three, some of the interpretative repertoires in operation around BLW are around a repertoire of the ‘agentive child’. We are also aware from the substantial research literature around maternal identities (e.g. Hays, 1996; Knaak, 2010) that mothers are orienting themselves as ‘good mothers’ in their parenting practices. Therefore, if we consider that there is a subject position of the ‘good mother’ at work here, we can consider this position in relation and with respect to the ‘agentive child’ repertoire. The way that this is demonstrated is that the data sets up BLW as being the informed choice to make for a good parent. Thus it follows that if you were adopting good parenting practices then you would make the decision to BLW, as the extract below suggests.

I was entirely focused on hunting down the recipe for Perfect Motherhood, determined to follow it to the letter. Co-sleeping, baby-led weaning, skin-to-skin contact, lots of fresh air and classical music; really, it was very simple. (Sunday Independent, Dublin, 6 October 2013)

This extract demonstrates that BLW is noted as one of the markers of a ‘good mother’ identity in our contemporary parenting culture and therefore opens up a ‘subject position’ for the mother who engages in these practices. Locke (2015) picks up this discussion in the context of ‘good mothering’ identities, BLW and parenting cultures further. Indeed, there are clear parallels between the breast/bottle debate and the decision women have around whether to spoon feed or do baby-led weaning demonstrated here. That is, to be positioned as a ‘good’ mother, women ought to take the baby-led approach. That said, participants have some agency with respect to subject positions and are able to adopt/resist them. Further, it is also possible to reposition themselves as a ‘good mother’ whilst not adopting BLW, through adopting other parenting practices. We will pick up an example of this in the extract below.

She wanted to give her children the very best start in life but in setting herself impossibly high standards, Leanne Morris came terrifyingly close to the edge … unaware of her condition, she pushed herself to be the perfect mum … ’After I had her we used real nappies and we did baby-led weaning where, instead of pureeing up her foods, we let her feed herself. We made sure we ate quite healthily –whatever we were eating we put down in front of Jessica. … So we were doing a few different things with Jessica but when John came along I couldn't cope with the pressure of it. (Daily Record, Glasgow, 2 July 2013)

In this extract we see how Leanne is positioning herself as a ‘good mother’ and wanting to do the best for her children. In contrast, BLW has been repositioned as being too demanding a method of feeding in some instances, in this case second time motherhood. Thus, by adopting it as a practice, she risks hers and her children’s wellbeing. Therefore to manage her ‘good mothering’ subject position, Leanne has positioned herself as only stopping BLW once she was unable to carry on due to her medical condition.

### Stage five: discursive accomplishments

In stage five, the focus should shift to the micro level of analysis to consider the action orientation of the discourse by examining the ways in which the accounts are put together in order to achieve particular interactional effects. That is, at this stage, the implications of discourse use are considered more locally in terms of what interactional business is being ‘done’ at the micro-level through the identification of linguistic and rhetorical devices. It is here that a CDP approach is able to utilise the ‘tool-box’ of discursive devices typical of a more conversation analytic inspired DP in order to consider how the discourse is constructed in particular ways. For example, strategies such as script formulations (Edwards, 1994) are identified which construct events as routine and ordinary; extreme case formulations (Pomerantz, 1986) which note when events have been constructed in extreme terms; and membership categories (Sacks, 1992), amongst others. In the following extract we will explore an example of the ways in which the extracts are constructed and the rhetorical tools at play in more detail. The piece is taken from the Daily Mail, a UK newspaper from 2013.

Put away the blender: the latest way to introduce food to your little darling is in chunks. No mush, no puree, no baby rice – just pieces of food, straight from your own plate. The method, coined baby-led weaning makes children less fussy and less stressed as well as healthier. Their kids eat – and wean – at their own pace from the age of six months, meaning the family can eat the same meal together (just keep it healthy). (The Daily Mail (UK), 20 February 2013)

The extract begins with a direct instruction – ‘put away the blender’ and continues with the ease of preparing food for BLW that it’s ‘just’ food ‘straight from your own plate’. This marks the routine nature of this method of weaning and food preparation. If we now start to break this down even further, BLW is discussed in terms of the effects on the child itself, presented in three parts; that it leads to children being ‘less fussy’ about the types of foods that they eat, ‘less stressed’ about eating, and ‘healthier’ given the foods that the children will select. This is an example of a ‘three part list’ – a rhetorical device initially identified by Jefferson (1990). A further example of this can be seen earlier to describe the ‘old’ ways of giving food – ‘no mush, no puree, no baby rice’. The three part list functions to bolsters the persuasiveness of an account and shores up the benefits of BLW as the (informed) best choice of weaning method. The extract finishes with another instruction to parents to ‘just keep it healthy’. The use of ‘just’ here, and at the start of the extract, is a discourse marker (Fraser, 1990; Schiffrin, 1987) that functions in this case as a hedging expression to minimise the force of the request given, yet also makes the request appear simple – something that any family should reasonably be able to achieve with little difficulty.

Similarly, if we revisit the example that demonstrated the repertoire of agentive child, we can demonstrate what a focus on the action orientation of the discourse accomplishes.

Babies may know best when it comes to their future health, according to researchers who found that infants who have more choice over what they eat may be less overweight than their spoon-fed counterparts. Allowing infants to feed themselves from a selection of finger foods from the start of weaning rather than being fed purees may help them regulate their intake. (The Guardian (UK) 7 February 2012)

The word ‘best’ here is an example of an extreme case formulation (Pomerantz, 1986). Extreme case formulations (ECF) work to bolster the strength and validity of the account, in this case, further persuading that BLW is the weaning method to adopt. There is a contrast noted between two categories of feeding behaviour – those who have been weaned in a baby-led fashion and those who are ‘spoon fed’. Here the categories are built up with types of behaviours that fit within them such as fussy/less fussy, convenient/less convenient and regulating intake/being spoon fed, and so on. The types of categories that are constructed here set up binaries that are working alongside a wider positioning of the ‘good mother’ who would make the ‘informed choice’ to adopt BLW. Note here also the softening use of ‘may’ throughout the piece on the article’s strong, agentive and extremely formulated claims (Edwards, 2000; Locke, 2004).

### Stage six: practice

We suggest the final stage involves putting together all of the different aspects of the analysis and considering what this means for the topic under investigation. Firstly, there is a consideration of what is achieved, in an ideological sense, by drawing on particular repertoires, and adopting certain subject positions whilst resisting others. These offer a macro level of analysis and enable the researcher to link the data to wider ideologies and societal discourses. For example, in the case of parenting and BLW, the move to natural, ‘permissive’ forms of parenting are having a renaissance in the parenting literature and contemporary parenting cultures, most notably through the attachment parenting ethos (Sears & Sears, 2001). However, as others (e.g. Badinter, 2012) have noted, this ‘overzealous’ approach to natural parenting sets up unattainable expectations and pressures for many mothers who are navigating parenting cultures. Therefore, we could argue that the repertoires and positions inherent in these discourses are both endorsed and resisted in the data presented here. The analytic stages presented in this paper demonstrate how BLW is constructed as the obvious ‘informed choice’ (Crossley, 2009: Kirkham, 2004) for the mother or parent to make. By adopting BLW, the infant becomes ‘agentive’ and able to self-regulate their feeding This then becomes packaged in the discourse with improved family relations and less stressed mealtimes, less ‘fussy-eating’ and potential healthier outcomes for the infant long-term. As Locke (2015) notes elsewhere, the construction of BLW as the ‘informed choice’ is reflective of both wider considerations of contemporary parenting cultures (Hays, 1996; Lee, Bristow, Faircloth, & McVarish, 2014) and neoliberal approaches to health promotion which operates on a system of informed choice and risk. When making these ‘choices’, the parent becomes accountable for the decisions that they make, particularly if they choose what is deemed to be the riskier option. In this final stage of analysis, we are able to put together the analytic jigsaw to demonstrate what the CDP analytic approach offers to the area of infant feeding. We can see that it has enabled a more in-depth consideration of the discourse around infant feeding decisions, setting these within wider contexts such as contemporary parenting ideologies and parenting cultures. By doing so we can begin to offer insight into determining how advice and choices, in terms of parenting practices, are both presented to, and made by, parents.

## Summary

This paper has set out to describe how to conduct a CDP analysis. It began with locating CDP within a wider discursive framework before outlining what it can offer health psychology research using baby led weaning as an example. As we noted previously, CDP enables the analysis to address the dual concerns of discourse; that is a focus on macro-level issues that consider wider societal discourses and the repertoires and subject positions inherent in these, combined with a focus on the micro, rhetorical and agentive aspects of the discourse. CDP offers a way of accessing these two levels of analysis and can ask, for example, what is at work in contemporary culture at this time that allows these particular versions to make sense? In the case of the example here, what do various constructions of baby-led weaning tell us about what parents are being advised to do, and the ‘preferred choices’ to be made, in terms of infant feeding practices? As we have seen the data demonstrates the repertoire of ‘the agentive child’. This became tied with a wider discourse of ‘good mothering’ and the subject position of the ‘good mother’ as one who adopted BLW given its convenience and benefits to the child. The analysis noted how the discourse was drawing on wider parenting ideologies, e.g. intensive mothering (Hays, 1996) that becomes evident in the data and link to wider societal norms around parenting and infant feeding. It also examines the ideological function of the discourse and,in this context of parenting cultures, there was a consideration of the kinds of parenting practices that were being portrayed as the preferred or ‘right’ way to parent.

Due to the situated nature of the analysis, CDP work, as with many other qualitative methodologies, does not attempt to generalise its findings beyond the data. This is not a shortcoming and it does not mean, however, that comparisons between data sets cannot be made. Work within the areas of Critical Discursive Psychological analysis explores discursive constructions and patterns that are recognisable in other wider contexts, different interactions and topic areas. For example, as we demonstrated in the analysis in this paper, there are particular recognisable ways of speaking around infant feeding and parenting practices that are relatable to the wider literature (e.g. Knaak, 2010; Locke, 2009; Stanway, 2005). As Wetherell (2015, p. 321) reminds us, discursive research and, in this case CDP, has provided critical and health psychology ‘with sets of tools, theories and method to systematically investigate’ areas of concern or contention within psychology and health to reach a wider and more contextualised understanding of pertinent issues. This paper has taken a step towards, and developed further, the CDP as a methodological approach to fulfil this important task.
